# Association between difficulty initiating sleep in older adults and the combination of leisure-time physical activity and consumption of milk and milk products: a cross-sectional study

**DOI:** 10.1186/1471-2318-14-118

**Published:** 2014-11-18

**Authors:** Naruki Kitano, Kenji Tsunoda, Taishi Tsuji, Yosuke Osuka, Takashi Jindo, Kiyoji Tanaka, Tomohiro Okura

**Affiliations:** Doctoral Program in Physical Education, Health and Sport Sciences, Graduate School of Comprehensive Human Sciences, University of Tsukuba, 1-1-1 Tennodai, Tsukuba, Ibaraki, 305-8574 Japan; Research Fellow of the Japan Society for the Promotion of Science, 5-3-1 Kojimachi, Chiyoda-ku, Tokyo, 102-0083 Japan; Physical Fitness Research Institute, Meiji Yasuda Life Foundation of Health and Welfare, 150 Tobuki, Hachioji, Tokyo, 192-0001 Japan; Gerontology Research Center, University of Jyväskylä, PO Box 35, Jyväskylä, 40014 Finland; Faculty of Health and Sport Sciences, University of Tsukuba, 1-1-1 Tennodai, Tsukuba, Ibaraki, 305-8574 Japan

**Keywords:** Exercise, Dairy, Insomnia, Elderly

## Abstract

**Background:**

Research has shown that engaging in leisure-time physical activity (LTPA) and consuming dairy foods can lead to better sleep. Combining these two non-invasive prescriptions may be more effective for helping people fall asleep. This study investigates whether participating in LTPA in conjunction with consuming milk and milk products has a beneficial association with difficulty initiating sleep (DIS) among older adults.

**Methods:**

The present study looked at 421 community-dwelling older people aged 65 years and older living in Ibaraki prefecture, Japan (mean age 74.9 ± 5.5 years, male 43.7%). We measured LTPA and sleep latency with the Physical Activity Scale for the Elderly and the Pittsburgh Sleep Quality Index, respectively. Participants who needed 30 minutes or more to fall asleep were defined as having DIS. We assessed dairy consumption as participants’ habitual intake of milk, yogurt and cheese.

**Results:**

After adjusting for covariates, participants who engaged in sufficient levels of LTPA as well as consumed milk (OR = 0.27, 95% CI = 0.10-0.73) or cheese (OR = 0.34, 95% CI = 0.14-0.85) were less likely to complain of DIS compared with people who neither engaged in LTPA nor ingested milk or cheese.

**Conclusions:**

Our findings suggest that the combination of engaging in LTPA and consuming milk or cheese is necessary as a prescription to improve falling asleep for older adults suffering from DIS. Additionally, engaging in LTPA along with dairy consumption may effectively improve a problem with falling asleep.

## Background

As people age, the quality and quantity of their sleep often deteriorate, and sleep complaints are extremely common in older adults. In a previous study approximately one out of three Japanese older adults reported insomnia symptoms [1]. Difficulty initiating sleep (DIS), difficulty remaining asleep, waking up early in the morning and lack of satisfying sleep are known as insomnia symptoms [2], with DIS being one of the most common symptoms in older people. Chronic DIS leads to high risks of mortality [3], diabetes [4], depression [5], poor physical function [6] and cognitive impairment [7]. Finding ways for older adults to improve their ability to fall asleep is essential to their well-being.

It has been established that regular leisure-time physical activity (LTPA), including exercise, has a beneficial effect on a person’s physical and psychological health [8, 9]. Recently, habitual LTPA is being recognized as a potentially effective factor for treating or preventing sleep problems; it is considered a non-pharmacological intervention to improve sleep. Previous randomized control trials (RCTs) [10, 11] have revealed that participating in LTPA decreases sleep latency (the time required to fall asleep). Furthermore, an epidemiological study [12] showed a relationship between engaging in exercise and a lower prevalence of DIS in older adults.

Primarily in western countries, people have consumed milk and milk products before bedtime as an effective means of improving their sleep. In fact, dairy products contain nutrients, particularly, tryptophan, a precursor to serotonin and melatonin, which play an important role in promoting sleep, including falling asleep [13]. Therefore, ingesting milk and milk products has been considered helpful in improving sleep, and a previous RCT reported that sleep efficiency is increased by melatonin-rich nighttime milk consumption in older people [14]. Since engaging in LTPA and consuming milk and dairy products are beneficial agents for sleep promotion, combining these two non-invasive prescriptions may more effectively help older adults suffering from DIS. However, there is limited literature on the relationship between sleep in older adults and the combination of these two daily activities. This study has two objectives: confirm independent associations between DIS and engaging in LTPA or consuming milk and milk products, and to examine whether combining these two habits is more strongly associated with a lower prevalence of DIS.

## Methods

### Data collection

A total of 437 community-dwelling, older Japanese individuals participated in the survey. The participants, aged 65 years and older, were recruited in Tsuchiura and Kasama Cities in Ibaraki prefecture, Japan in 2013. The Tsuchiura City participants consisted of 160 community dwellers (mean age 75.6 ± 6.2 years, male 34.0%) recruited through local advertisements and flyers or invited by municipal employees from June to November 2013. For Kasama City, we used data from 277 randomly selected individuals (mean age 74.5 ± 5.0 years, male 49.6%) who participated in the “Kasama study” [15] from July to August 2013. We started with 437 participants, but we rejected 16 individuals due to incomplete data. There were 421 final participants (mean age 74.9 ± 5.5 years, male 43.7%) in the data analysis. All participants provided a signed informed consent. The Ethics committee of the University of Tsukuba approved this study.

### Leisure-time physical activity

To assess the amount of LTPA, we used the Japanese version of the Physical Activity Scale for the Elderly (PASE) [16]. A previous study deemed the PASE to be reliable and valid [16]. LTPA contains five activities: walking (for both recreation and transportation); light-, moderate-, and vigorous-intensity recreational activities; and muscle strength training. We measured a participant’s frequency (day/week) and duration (minute/day) of each activity during the previous 7 days and calculated a total LTPA score using frequency, duration and weight for each activity used in the analysis.

### Milk/milk products consumption

We assessed the consumption of milk, yogurt and cheese during the previous couple months using a self-reported questionnaire and the following questions: “How many days a week do you drink milk (or eat yogurt/cheese) [day (s)/week]?” and “How much milk (or yogurt/cheese) do you usually consume each day [milliliters or grams/day]?” Additionally, we supplied examples of portion size (e.g. a glass of milk = 200 ml) to help participants answer the questions. Using these two questions, we calculated consumption of each food item, and the sum of the three items equaled a participant’s total dairy consumption used in the analysis. To estimate total dairy consumption, units of milk intake were converted from milliliters to grams using the following equivalency: 100 ml = 103.2 gm” [17]. No participants had a milk allergy. Previous studies have used self-reported questionnaires to evaluate the frequency and the portion size of daily milk and milk product consumption; those authors confirmed a moderate correlation (r = 0.4–0.6) with the weighed dietary recording considered the gold standard for assessing dietary methods [18, 19].

### Sleep

We gathered information on total sleep time and sleep latency during the previous month and evaluated these data by referencing the Pittsburgh Sleep Quality Index (PSQI) [20]. PSQI is used to identify sleep disorders in both clinical and research fields throughout the world, and validity and reliability have been confirmed [20].

### Potential confounders

We referenced previous studies to determine minimum required potential confounders for our study: age, gender, body mass index (BMI), hypnotic use for sleep [21], engaging in LTPA [22] and dairy consumption [23, 24]. We obtained age, gender and hypnotic use with a self-report questionnaire. We measured height and weight with a stadiometer or a digital weight scale and calculated BMI calculated as weight in kg divided by height in meters squared.

### Statistical analysis

LTPA participation was divided into 3 groups based on median level of engagement or no engagement at all: 1) nothing, 2) low level and 3) high level. Total dairy consumption was divided into 3 groups using tertiles: 1) low level, 2) moderate level and 3) high level. A variable composed of the following 4 groups combining LTPA and intake of dairy products was created: 1) LTPA with large dairy intake, 2) LTPA with small dairy intake, 3) no LTPA with large dairy intake and 4) no LTPA with small dairy intake. By referencing previous studies [25], people who took more than 30 min to fall asleep were defined as having DIS.

We analyzed group differences (non-DIS vs. DIS), using independent *t*-test and chi-square test. We used logistic regression analysis to examine the association between participation in LTPA, consumption of dairy products or a combination of those two variables (dependent variables) with DIS (independent variable) adjusting for age, gender, hypnotic use, BMI and those LTPAs or dairy products which were not used as dependent variables. In addition to the odds ratios, we also calculated *P* for trend. Statistical analysis was performed by IBM SPSS Statistics version 19.0 with the level of significance set at *P* < 0.05.

## Results

### Description of the study participants

The characteristics of study participants are shown in Table 1. The mean age was 74.9 ± 5.5 years, and the subjects’ usual sleep time was 405.9 ± 73.4 min/night. Out of 421 participants, 135 individuals met the definition of DIS (32.1%) and 80 people (19.0%) habitually used sleeping pills. Compared with participants who did not have DIS, individuals defined as having DIS were more likely to be women, experienced shorter total sleep duration and long sleep latency, engaged less in LTPA and were more likely to use hypnotics (*P* < 0.05).

**Table 1:** **Characteristics of study participants**

	All (n = 421)	Non-DIS (n = 286)	DIS (n = 135)
	Mean ± SD	Mean ± SD	Mean ± SD
Age, years	74.9 ± 5.5	74.7 ± 5.5	75.3 ± 5.6
Women, n (%)	237 (56.3)	149 (52.1)	88 (65.2)*
Height, cm	155.2 ± 9.0	156.0 ± 8.6	153.6 ± 9.5*
Weight, kg	56.1 ± 9.6	56.9 ± 9.7	54.4 ± 9.3*
Body mass index, kg/m^2^	23.2 ± 2.9	23.3 ± 2.9	23.0 ± 2.8
Total sleep time, min	405.9 ± 73.4	411.6 ± 70.8	393.7 ± 77.3*
Sleep latency, min	21.1 ± 21.5	10.7 ± 5.5	43.2 ± 25.8*
Hypnotic use, n (%)	80 (19.0)	34 (11.9)	46*
Leisure-time physical activity, points	21.7 ± 25.1	23.7 ± 26.9	17.6 ± 20.5*
*Milk consumption*			
Frequency, days/week	3.6 ± 2.9	3.8 ± 3.0	3.2 ± 2.9
Volume, ml/day	155.9 ± 127.1	162.9 ± 129.4	141.0 ± 121.1
Total consumption, ml/week	784.3 ± 813.4	819.0 ± 833.1	710.9 ± 767.7
*Yogurt consumption*			
Frequency, days/week	4.0 ± 2.8	3.9 ± 2.9	4.1 ± 2.8
Volume, g/day	104.6 ± 79.0	100.6 ± 72.2	113.4 ± 91.9
Total consumption, g/week	506.6 ± 491.4	469.9 ± 418.7	587.1 ± 616.0*
*Cheese consumption*			
Frequency, days/week	1.3 ± 1.8	1.3 ± 1.8	1.3 ± 2.0
Volume, g/day	14.3 ± 16.6	15.6 ± 17.3	11.5 ± 14.8*
Total consumption, g/week	35.6 ± 55.3	37.4 ± 56.6	31.9 ± 52.6
*Total dairy consumption*			
Frequency, days/week	5.4 ± 2.4	5.4 ± 2.4	5.3 ± 2.3
Volume, g/day	193.9 ± 149.4	194.2 ± 145.0	193.5 ± 159.0
Total consumption, g/week	1357.5 ± 1045.9	1359.1 ± 1015.0	1354.3 ± 1112.9

*P < 0.05 (Non-DIS vs. DIS).

DIS: difficulty initiating sleep (30 min **≤** sleep latency).

SD: standard deviation.

### LTPA and DIS

An association between LTPA and DIS is presented in Table 2. After adjusting for age, gender, hypnotic use, BMI and consumption of milk and milk products, a high level of LTPA was associated with decreased prevalence of DIS compared to no engagement in LTPA (OR = 0.48, 95% CI = 0.25-0.93).

**Table 2:** **Association between LTPA and DIS**

	DIS		
	n(%)	Odds ratio	95% CI
*Leisure-time physical activity*		Trend *P* value = 0.076	
Not engaging	57 (14.4)	1.00	
Low level (0.1–16.9 points)	168 (42.3)	0.72	0.38–1.37
High level (≥17.0 points)	172 (43.3)	**0.48**	**0.25–0.93**

Bold numbers indicate *P* < 0.05.

DIS: difficulty initiating sleep (30 min **≥** sleep latency); LTPA: leisure-time physical activity; 95% CI: 95% confidence interval.

Odds ratios and 95% confidence intervals were adjusted for age, gender, hypnotic use, body mass index and consumption of milk and milk products.

### Milk/milk products and DIS

As shown in Table 3, participants were less likely to perceive DIS when they consumed a high level of milk (OR = 0.47, 95% CI = 0.26-0.84) even after adjusting for age, gender, hypnotic use, BMI, LTPA engagement and yogurt and cheese intake. Moreover, a significant dose–response relationship was observed between milk intake and prevalence of DIS (Trend *P* < 0.05). There were no significant associations between consumption of yogurt, cheese or total dairy and DIS.

**Table 3:** **Association between milk and milk products consumption and DIS**

	DIS			
	n(%)	Odds ratio	95% CI	
*Milk consumption*		**Trend** ***P*** **value = 0.041**		
Not consuming	105 (26.4)	1.00		
Low level (0–1049 ml/w)	149 (37.5)	0.66	0.37–1.18	
High level (≥1050 ml/w)	143 (36.0)	**0.47**	**0.26–0.84**	
*Yogurt consumption*		Trend *P* value = 0.554		
Not consuming	72 (18.1)	1.00		
Low level (0–599 g/w)	164 (41.3)	0.98	0.51–1.87	
High level (≥600 g/w)	161 (40.6)	1.27	0.66–2.45	
*Cheese consumption*		Trend *P* value = 0.089		
Not consuming	179 (45.1)	1.00		
Low level (0–49 g/w)	115 (29.0)	0.58	0.33–1.01	
High level (≥50 g/w)	103 (25.9)	0.61	0.34–1.08	
*Total dairy consumption*		Trend *P* value = 0.242		
Low level (0–805 g/w)	133 (33.5)	1.00		
Moderate level (806–1605 g/w)	132 (33.2)	1.22	0.71	2.10
High level (≥1606 g/w)	132 (33.2)	1.22	0.43	1.34

Bold numbers indicate *P* < 0.05.

DIS: difficulty initiating sleep (30 min **≥** sleep latency); 95% CI: 95% confidence interval.

Odds ratios and 95% confidence intervals were adjusted for age, gender, hypnotic use, body mass index, leisure-time physical activity and consumption of milk and milk products that were not used as independent variables.

### LTPA with milk/milk products and DIS

As Table 4 indicates, after controlling for age, gender, hypnotic use, BMI and yogurt and cheese consumption, participants who engaged in LTPA as well as consumed milk were less likely to complain of DIS compared with those who neither engaged in LTPA nor consumed milk (OR = 0.27, 95% CI = 0.10-0.73). Similarly, after adjusting for multiple confounding variables, we found that the combination of LTPA engagement and cheese consumption was associated with decreased prevalence of DIS (OR = 0.34, 95% CI = 0.14-0.85). We did not find a significant association between DIS and the combination of LTPA and yogurt or total dairy consumption.

**Table 4:** **Associations between DIS and LTPA along with milk and milk products consumption**

			DIS		
			n(%)	Odds ratio	95% CI
*LTPA*		*Milk*			
Not engaging	×	Not consuming	19 (4.8)	1.00	
Not engaging	×	Consuming	38 (9.6)	0.42	0.13–1.37
Engaging	×	Not consuming	86 (21.7)	0.45	0.16–1.30
Engaging	×	Consuming	254 (64.0)	**0.27**	**0.10–0.73**
*LTPA*		*Yogurt*			
Not engaging	×	Not consuming	14 (3.5)	1.00	
Not engaging	×	Consuming	43 (10.8)	1.08	0.30–3.96
Engaging	×	Not consuming	58 (14.6)	0.57	0.16–2.06
Engaging	×	Consuming	282 (71.0)	0.64	0.20–2.03
*LTPA*		*Cheese*			
Not engaging	×	Not consuming	26 (6.5)	1.00	
Not engaging	×	Consuming	31 (7.8)	0.66	0.21–2.09
Engaging	×	Not consuming	153 (38.5)	0.59	0.24–1.45
Engaging	×	Consuming	187 (47.1)	**0.34**	**0.14–0.85**
*LTPA*		*Total dairy*			
Not engaging	×	Low level (0–12933 g/w)	32 (8.1)	1.00	
Not engaging	×	High level (≥12934 g/w)	25 (6.3)	1.90	0.62–5.82
Engaging	×	Low level (0–12933 g/w)	167 (42.1)	0.83	0.36–1.91
Engaging	×	High level (≥12934 g/w)	173 (43.6)	0.77	0.33–1.76

Bold numbers indicate *P* < 0.05.

DIS: difficulty initiating sleep (30 min **≥** sleep latency); LTPA: leisure-time physical activity.

Odds ratios and 95% confidence intervals were adjusted for age, gender, hypnotic use, body mass index and consumption of milk and milk products that were not used as independent variables.

## Discussion

We found that higher levels of LTPA and greater milk consumption were each associated with lower prevalence of DIS in older adults. Furthermore, we found that combining LTPA participation and milk and/or cheese consumption was more favorably linked with lower prevalence of DIS.

### Description of the study participants

In the present study, complaints of DIS were reported by 37.1% of women and by 25.5% of men, which is similar to other Japanese studies [1]. Furthermore, since a large body of evidence also indicates there is a higher prevalence of insomnia in older women than in older men [26], the rates of insomnia in the study participants do not appear to be specific to our study population.

### LTPA and DIS

In this study, sufficient LTPA was associated with a lower prevalence of DIS, which is in accord with a large amount of research showing that exercise improves sleep latency [10, 11, 27] and physically fit individuals have shorter sleep latency [28]. Although the mechanisms are not clear, earlier studies have suggested that physical activity may improve sleep due to 1) body temperature increases, 2) energy expenditure, 3) antianxiety and antidepressant effects and 4) entrainment of circadian rhythm [22, 29]. The improvement we saw in DIS in our study may be a consequence of these physiological and psychological effects of LTPA.

### Milk/milk products and DIS

We found a positive link between decreased prevalence of DIS and the intake of milk, but not yogurt, cheese or total dairy consumption. At present, there are divergent opinions as to the effects of milk on sleep; some researchers found that consuming milk improves sleep quality [30, 31], whereas, others reported no significant effects of milk intake [14, 32]. Yamamura et al. demonstrated a significant improvement in sleep efficiency after ingesting melatonin-rich milk before bedtime [14]. From results of our study, we suggest that daily intake of ordinary commercial milk may also facilitate falling asleep. Our findings confirmed the beneficial effect that commercial milk consumption can have on the prevalence of DIS, which is useful information for the older adult population.

We also found a significant linear trend between drinking milk and a lower prevalence of DIS, that is, DIS became less frequent as the amount of milk consumed increased. To our knowledge, literature concerning the association between sleep and milk/milk products intake in older people is limited. In particular, the dose–response relationship between these factors is still unclear. Hence, our findings contribute to this developing area of research.

### LTPA with milk/milk products and DIS

To our knowledge, although some studies have investigated the relationship between sleep and either LTPA or milk/milk products, no prior study has evaluated the association between DIS and LTPA in conjunction with dairy intake. Our research produced interesting and novel findings as it shows that older adults who engaged in LTPA and consumed milk or cheese reported DIS less frequently than people who neither engaged in LTPA nor consumed milk or cheese. Additionally, individuals who only engaged in LTPA or only consumed milk or cheese had higher odds ratios than people with both habits. The combination of LTPA and milk or cheese consumption may be an effective tool to help people fall asleep.

In modern society, older adults frequently complain of insomnia symptoms including difficulty falling asleep [1]. They may turn to medication to attain sleep, although medications have a greater risk of side effects e.g. falling [33] and even death [34]. Attele et al. showed that older people are often reluctant to take sleeping pills because of the negative impacts on their health [35], hence, non-pharmacological therapy with less adverse effects is needed. Along with the beneficial effects on sleep, LTPA provides other health benefits such as maintaining physical function [36] and decreased risks of mortality [37] and dementia [38]. Milk and milk products also contribute significant amounts of calcium, which plays an important role in reducing risks of osteoporosis [39] and hypertension [40]. Due to these benefits, participating in LTPA and consuming milk or dairy foods are recommended for well-being in later life. Furthermore, taking into account our findings on sleep improvement, we suggest that habitual LTPA along with milk or cheese consumption are helpful not only for maintaining general health, but also as a non-drug intervention for DIS in older adults.

### Limitations

There are some limitations in our study. First, the generalization of the study’s results is uncertain. Almost all study participants entered the study after receiving an invitation letter or seeing a local advertisement. Thus, the study may underestimate the proportion of older adults with poor living habits (e.g. inactive, low consumption of dairy products or insomnia) or those in poor health. Additionally, sample size was small, specifically with regards to people who did not participate in LTPA and/or consume dairy products; this limits confidence in the results. To verify our results, future investigations with larger sample sizes in unhealthy, older adults are needed. Second, we did not assess depressive symptoms, cognitive function/dementia, chronic illness (e.g. heart disease or lung disease) [41, 42] or a number of medications [43] which could be potential confounders for our results. Third, since LTPA, dairy consumption and sleep were assessed via self-reported questionnaires, there may be recalling/reporting bias in our data. In particular, Silva et al. suggested that sleep latency might be overestimated by a self-reported measure [44], thus, future studies should include objective measures of sleep, such as the use of actigraphy. Fourth, because of the present study’s cross-sectional design, we could not prove a causal relationship. There may be a bidirectional relationship between physical activity and sleep [45, 46]. To reveal a causal relationship, a prospective cohort study and an intervention study are needed. Finally, unfortunately, we did not assess the times of day that participants’ practiced LTPA and consumed milk/dairy products. The effect that exercise has on initiating sleep differs depending on when it was performed [22]. Similarly, ingesting milk and other milk products, which contain several nutrients with potential sleep-promoting properties, may be effective for falling asleep when these food items are consumed before bedtime.

Because earlier studies have revealed that LTPA and dairy intake separately have positive effects on DIS, it is reasonable to conclude from our findings that LTPA in combination with milk or cheese intake may help decrease DIS, even with the above study limitations.

## Conclusions

We studied the association between LTPA in conjunction with milk or other dairy products intake and DIS in older adults. Our data revealed that higher levels of LTPA related to a lower prevalence of DIS and decreased complaints of difficulty falling asleep, in a dose–response relationship, with consumption of milk. Moreover, it is worth mentioning that people who engage in LTPA and drink milk or eat cheese may be falling asleep easily in contrast to those who do not engage in either of those activities. This information could be useful in the development of a non-pharmacological intervention for insomnia and to help promote sleep quality in older people.

## Abbreviations

DIS: Difficulty Initiating Sleep
LTPA: Leisure-time Physical Activity
BMI: Body Mass Index
PSQI: Pittsburgh Sleep Quality Index.
